# The Role of NT-proBNP Levels in the Diagnosis and Treatment of Heart Failure with Preserved Ejection Fraction—It Is Not Always a Hide-and-Seek Game

**DOI:** 10.3390/jcdd11070225

**Published:** 2024-07-16

**Authors:** Christina Chrysohoou, Konstantinos Konstantinou, Kostas Tsioufis

**Affiliations:** 1st Cardiology Clinic, Hippokration Hospital, National and Kapodistrian University of Athens, 11527 Athens, Greece; kostikon@gmail.com (K.K.);

**Keywords:** heart failure, NPs, HFpEF, diet, NT-proBNP

## Abstract

Although heart failure with preserved ejection fraction (HFpEF) has become the predominant heart failure subtype, it remains clinically under-recognized. This has been attributed to the complex pathophysiological mechanisms that accompany individuals with several co-morbidities and symptoms and signs of HFpEF. Natriuretic peptides have been recognized as playing an important role in the diagnosis and monitoring of patients with heart failure with reduced ejection fraction (HFrEF), but their role in HFpEF remains controversial, driven by the different pathophysiological characteristics of these patients. The type of diet consumed has shown various modifying effects on plasma levels of NPs, irrespective of pharmacological treatment.

## 1. Introduction

Heart failure with preserved left ventricular ejection fraction (HFpEF) represents a complex pathophysiological condition, diagnosed in almost half of patients with heart failure symptoms, while sharing the same prognosis with heart failure with reduced ejection fraction (HFrEF). Research in the last decade has substantially advanced the understanding of the pathophysiology of HFpEF [1,2]. However, diagnosis in these patients remains challenging, especially compared to HFrEF. Natriuretic peptide (NP) measurement by general practitioners in individuals from high-risk populations, such as those with arterial hypertension or type 2 diabetes mellitus (T2DM), may help in identifying patients with elevated ventricular diastolic pressure. This will hasten the initiation of preventive measures, including medicine up-titration or novel therapies, therefore preventing or slowing down the progression of heart failure [3,4]. In the case of patients with comorbidities such as obesity, an HFpEF diagnosis may be underestimated due to the limitations of NP secretion and resting echocardiography measurements [5,6]. In this review, we aimed to present the current knowledge on the role of N-Terminal Pro-B-Type Natriuretic Peptide (NT-proBNP) in the diagnosis and monitoring of patients with HFpEF to illustrate pathophysiologic explanations that justify limitations in their use.

## 2. Pathophysiology of HFpEF

Heart failure with preserved left ventricular ejection fraction does not represent a rare clinical condition. Nowadays, the definition of heart failure does not depend on left ventricular ejection fraction, as even patients with signs and symptoms of congestion may show a small left ventricle cavity size (sometime with increased wall thickness), or significantly impaired long axis function with preserved ejection fraction, which accompanies low cardiac output. Thus, heart failure can be presented with normal, increased, or reduced end-diastolic volume, the driving force of reduced stroke volume. HFpEF has been described as a condition resulting from complex pathophysiological procedures including abnormalities in peripheral blood circulation, coronary microvascular dysfunction, chronotropic incompetence, intrinsic left ventricular systolic dysfunction, pericardial restraint, and vascular stiffening [7,8]. Patients with HFpEF exhibit diastolic dysfunction due to impaired relaxation and/or increased passive stiffness, and imaging reveals left atrial dysfunction accompanied by right ventricular systolic impairment and progressive pulmonary hypertension. In a simplified approach to HFpEF assessment, four main phenotypes have been introduced: the stiff artery HFpEF phenotype, where decreased aortic compliance with decreased vasorelaxation with exercise and decreased blood pressure lability lead to alterations in arterio-ventricular coupling, causing impaired left ventricular function; obese HFpEF, where fat accumulation in the epicardial space promotes systemic inflammation, plasma expansion, and insulin resistance, leading to bi-ventricular remodeling, volume overload, and pericardial restraint; left atrial myopathy, where increased left ventricular filling pressures lead to morphologic and functional alterations of the left atrium, causing alterations to the pulmonary circuit and right ventricular dysfunction; and ischemic HFpEF, where macrovascular or microvascular ischemia causes alterations in the left ventricular systolic and diastolic function [9,10]. In clinical practice, most patients with HFpEF show a combination of these phenotypes, where several pathophysiological procedures overlap and cause differing expressions of circulating biomarkers and cardiac imaging results [11]. 

## 3. Clinical Course of HFpEF

HFpEF incidence is rising relative to HFrEF, exhibiting a lifetime risk of 1 out of 10 individuals at the age of 45 years old. The prevalence of HFpEF is highly age-dependent, with a 5-year mortality of 75% and a 30-day all-cause readmission rate of 21%; these findings are quite similar to those for HFrEF (ref). Several co-morbidities that accompany HFpEF or play a part in its pathophysiological process have an important role in its clinical course. The progression of HFpEF across all spectra of phenotypes occurs over a long period of time. At the initial stage of HFpEF, there is relatively little cardiac remodeling and mild impairment in the left ventricle mechanics, with an elevation in left ventricle filling pressure only occurring during exercise. With time, there is progressive deterioration in the left atrial function, and remodeling occurs, leading to secondary atrial functional mitral regurgitation and the development of paroxysmal atrial fibrillation. At this stage, mild elevation in the left ventricle filling pressure at rest may develop. With the further progression of HFpEF, the left atrial remodeling and, consequently, dysfunction become more evident, and permanent atrial fibrillation may occur. The main observations seen in advanced HFpEF are the worsening of pulmonary hypertension, pulmonary vascular remodeling, and vasoconstriction, while right ventricle and right atrial dysfunction contributes to reduced preload and severely impaired cardiac output. Increased pericardial restraint can also contribute to advanced heart failure symptomatology [1,2,12,13,14]. The clinical course of HFpEF is underpinned by hemodynamic and cellular/molecular mechanisms. The cellular mechanisms include structural changes in cells and the widening of the extracellular space as well as cardiometabolic alterations due to differences in the main source of energy handling, leading to microvascular inflammation. In parallel, there is ongoing activation of hemodynamic mechanisms with left ventricular diastolic dysfunction, leading to post-capillary pulmonary hypertension, tricuspid valve insufficiency, and right ventricular dysfunction in combination with pulmonary vascular disease. In such advanced stages, an increased transpulmonary gradient, as measured using cardiac catheterization, is a marker of combined post- and pre-capillary pulmonary hypertension. 

Thus, one of the main challenges is the early recognition of the occurrence of HFpEF using a non-invasive method with high accuracy in discriminating symptoms related to HFpEF from those of other conditions.

## 4. The Role of NT-proBNP in the Diagnosis of HFpEF

For the diagnostic workup of HFpEF, two algorithms have been created by the American College of Cardiology and the European Society of Cardiology. The first one, which is more simplified, uses clinical factors like age, obesity, arterial hypertension, atrial fibrillation, pulmonary hypertension, and evidence of diastolic dysfunction, while the second uses functional and morphological indices from echocardiography and levels of NT-proBNP. Both scores, according to the points aligned to each characteristic, categorize patients as having a low, intermediate, or high probability of having HFpEF [15,16]. In contrast to the diagnostic algorithm used for HFrEF, in HFpEF, the NT-proBNP and BNP measurements do not play a sole role, even as a high-specificity marker. Interestingly, in HFpEF patients, NT-proBNP values do not always direct diagnosis, as a significant proportion (up to 20–25%) show low BNP/proBNP (NP) levels, even with confirmed increased levels of pulmonary wedge pressures. This situation has been attributed to genetic factors; obesity, with mainly pericardial restraint due to adipose tissue, but with mild intrinsic myocardial involvement; a lack of wall stress; insulin resistance; and increased androgens. Nevertheless, even HFpEF patients with mild intrinsic myocardial involvement show a threefold worse outcome compared with patients without HFpEF [17,18]. 

What is the main pathophysiological explanation for this controversy?

The prime stimulus for the synthesis and release of BNP is myocyte stretch secondary to transmural distending pressure. On the cleavage of proBNP 108, NT-proBNP 1–76 is released in a 1:1 ratio with its carboxy-terminal congener BNP 1–32. Plasma levels of NPs are influenced by the left ventricle’s structure and function. In HFpEF, especially in the early stages, the observed increase in the left ventricular dimensions is smaller than the increase in wall thickness, which is influenced differently by internal ventricular dimensions, wall thickness, and interventricular pressure on unit wall stress and cardiomyocyte stretch compared to HFrEF. The primary driver of NP synthesis is the increased wall tension; thus, the diagnostic performance of NPs can be impaired, especially in the early stages of HFpEF. In the acute setting of advanced symptomatic HFpEF, NPs preserve their diagnostic role, but in the setting of incipient or treated HF, they may exhibit sub-diagnostic values [19,20]. Accordingly, the plasma NP levels in acute decompensated HF (ADHF) are lower in HFpEF than in HFrEF. 

Plasma NT-proBNP (>600 pg/mL) and BNP (>100 pg/mL) are strong, relatively nonspecific, independent predictors of left ventricular restrictive filling. In HF, plasma NT-proBNP correlates with E/e’, an echocardiographic index of LV filling pressure. Alongside right heart function, plasma concentrations of B-type NPs are inversely related to right ventricular ejection fraction and directly related to right ventricular dimensions and estimated intraventricular pressure. The PARAMOUNT trial of valsartan–sacubitril therapy in HFpEF demonstrated a significant relationship between plasma NT-proBNP and decreases in LV systolic longitudinal and circumferential strain, independently of age, sex, systolic and diastolic blood pressure, body mass index, left ventricular ejection fraction, left atrial volume index, atrial fibrillation, and renal function [21,22].

A recent secondary analysis of the RELAX trial evaluated the role of biomarkers corresponding to echocardiographic phenotypes in HFpEF. In this analysis, 216 patients with HFpEF were classified into three categories: those with phenotype A, who were characterized by moderate (grade I–II) diastolic dysfunction, low values of left atrial enlargement, the least amount of right ventricular dysfunction, and the highest left ventricular ejection fraction; phenotype B, which was the most common one, with the highest rate of grade III diastolic dysfunction, significant enlargement of the left atrium, elevated E/e’, and right ventricular dysfunction; and phenotype C, with little to no diastolic dysfunction, the second highest rate of left atrial enlargement, and normal E/e’ in transmitral velocity according to Doppler imaging. Interestingly, NT-proBNP was associated with increasing left atrial enlargement and an increased E/e’ ratio; it was evident in groups A and C, and was elevated mainly in those patients who presented with myocardial fibrosis [23]. 

### 4.1. Obesity and NT-proBNP Levels 

Obese women and men show an increased cumulative incidence of HFpEF compared to non-obese individuals. Obesity is also related to physical inactivity and increased blood volume that lead to HFpEF. Increased body weight promotes reduced contractility in the right ventricle and decreased myocardial efficiency, leading to increased myocardial stiffness. These pathophysiological alterations increase the release of NPs into the blood stream, although NP levels do not always reflect the severity of cardiac dysfunction in obese patients. This has been attributed to the increased degradation of NPs in lipid tissue, but also to the extracardiac accumulation of fat that leads to pericardial restraint, causing the inhibition of myocardial stretch [24].

### 4.2. Atrial Remodeling and NT-proBNP Levels

In patients with acute HF and atrial fibrillation, the levels of NT-proBNP may be lower than expected. The absence of left atrial contraction is associated with decreased left ventricular filling and the loss of atrioventricular coupling. This results in a reduction in cardiac output. When left atrial remodeling becomes advanced, the left ventricular preload is progressively impaired, and the loss of atrioventricular coupling and pericardial restraint results in the reduced secretion of NPs from ventricular myocytes [25].

Thus, the interpretation of the results of every test is important as, in the clinical setting of HFpEF with many comorbidities, NT-proBNP levels should be considered in concert with the clinical history, examination findings, and data from other tests, including a standard laboratory workup and cardiac imaging (Table 1). Anthropometric indices and clinical parameters, like age, obesity, preserved ejection fraction, renal dysfunction, and atrial fibrillation, may affect the diagnostic performance of NT-proBNP. 

## 5. Role of NT-proBNP in Risk Stratification of HFpEF

In the prognosis of HF patients, left atrial pressure, mitral valve filling, and right ventricle function play the most important roles [26]. Previous studies have revealed that in patients with exertional dyspnea and preserved left ventricular ejection fraction, the NT-proBNP levels at rest correlated with the mean wedge pressures at the peak of exercise [27,28]. In the context of the STRONG HF study, 15% of the patients had HFpEF. In the whole study group, intensified therapy, driven by NT-proBNP levels, demonstrated good tolerance and safety, and there was a reduction in the rehospitalization rate of 180 days, illustrating the need for personalized medicine and shared decision-making [29].

## 6. Preventing Heart Failure Occurrence at Stage A 

In the primary prevention of HF occurrence, evidence for the role of biomarkers in diabetic patients is obtained from the PONTIAC II (NT-proBNP Selected PreventiOn of cardiac eveNts in a populaTion of dIabetic patients without A history of Cardiac disease). In patients with NT-proBNP > 125 pg/mL without cardiac disease randomized into a “control” group and an “intensified” group (receiving up-titration of RAS antagonists and betablockers), a significant reduction in the primary endpoint (composite CV death and CV hospitalization) (HR: 0.351, *p* = 0.044) was observed in the intensified group [30,31]. 

Since, in diabetic cardiomyopathy, increased strain is imposed on the left ventricle and these peptides have been shown to correlate with transvalvular gradients and left ventricular hypertrophy, it would be reasonable to examine the possible use of natriuretic peptides for improving the prognosis of the time of symptom onset [32,33]. 

NPs have proven their value in the diagnosis of patients presenting with shortness of breath and in the clinical management of patients with heart failure. However, the long-term prognostic role of natriuretic peptides is being particularly studied in subjects with risk factors for HF development (ACC stage A) and not clinically overt HF. In a recent meta-analysis recruiting data from 40 studies with 95,617 individuals without a history of CV disease, NT-proBNP levels were found to serve as strong predictors for first-onset HF and ongoing coronary heart cardiomyopathy and stroke incidence. Furthermore, NT-proBNP levels showed higher incremental ability for coronary heart cardiomyopathy and stroke than HDL cholesterol or even C-reactive protein levels. This analysis illustrates that NT-proBNP could serve as a multipurpose biomarker, integrating HF into the context of primary prevention [34]. Additionally, in 16,492 patients with T2DM and a history of or at risk of cardiovascular events, high NT-proBNP levels were found to serve as predictors for future hospitalization for heart failure. The use of the established dichotomous cut point of 125 pg/mL for individuals aged beyond 75 years correlates with a significant increased risk of hospitalization for heart failure. Additionally, higher NT-proBNP concentrations above 400 pg/mL, are associated with a significantly heightened risk of CV events, as, in clinical practice, discriminating whether patients belong to high- or low-risk categories can be based in NT-proBNP concentrations ≥400 pg/mL and <400 pg/mL, respectively [35,36]. 

## 7. Medication and NP Levels

Medication therapy in HFpEF patients has not shown significant impact on NP levels. In the TOPCAT study, higher NT-proBNP levels were associated with a worse prognosis, but the treatment benefit was greatest in the lowest risk tertile (<682 ng/L) of NT-proBNP, where fibrosis was likely predominant [37]. Additionally, irbesartan in HF mildly reduced EF/HFpEF (I-PRESERVE) showed a progressive increase in morbidity and mortality with increasing plasma concentrations of NT-proBNP, but the medication did not improve overall prognosis [38]. In the case of empagliflozin in HFpEF, there was a borderline statistical difference on the impact of therapy on NT-proBNP levels, although the impact on clinical outcomes was more significant [39]. The PARALLAX study, in 2572 patients with HFpEF, treatment with sacubitril/valsartan versus standard medical therapy showed no effect on plasma NT-proBNP levels or the 6-min walking test [40]. Recently, semaglutide treatment led to significant improvements in exercise capacity, as measured by the 6 min walk distance and weight balance, compared to the placebo group, but with no significant effects on NT-proBNP levels [41]. 

## 8. Dietary Factors in HFpEF and NP Levels 

The role of diet has been extensively investigated in general health [42]. Recent studies have also explored the role of diet on the hemodynamic conditions of the heart, including biomarkers related to inflammation and elevated filling pressure. In 9782 adults from the NHANES 1999–2004 study without self-reported cardiovascular disease, a higher-quality diet, specifically a lower dietary intake of sodium and added sugar, was associated with lower serum levels of NT-proBNP [43]. Several clinical trials have demonstrated that higher adherence to a Mediterranean or DASH diet is related to lower blood levels of NT-proBNP. In the Prevenzion con Dieta Mediterranean (PREDIMED) trial, which enrolled individuals at a high risk of cardiovascular disease, those consuming a traditional Mediterranean diet had lower NT-proBNP levels compared to those assigned to a low-fat diet. In the DASH trial, individuals who were randomly assigned to the DASH diet intervention for 8 weeks had lower levels of NT-proBNP than those who followed a control diet, such as a typical American diet; this was mainly attributed to the diet’s beneficial effects on oxidative stress and inflammation. Excessive salt intake induces inflammation and seems to produce reactive oxygen species and activate the transcription of mineralocorticoid-receptor-dependent genes, which can raise circulating NP levels [44,45]. Furthermore, salt also activates the renin–angiotensin–aldosterone system, leading to increased fluid retention and volume expansion, which can place a strain on the heart and blood vessels and, thus, elevate NT-proBNP [46].

The Mediterranean type of diet has exhibited beneficial effects in many biochemical and clinical parameters. A closer adherence to a healthy dietary pattern, in the concept of PREDIMED, has been related to lower cardiac mass, reflecting lower wall stress and less secretion of NPs, in parallel with better control of other comorbidities like obesity, arterial hypertension, and hyperinsulinemia [47,48]. Healthy dietary patterns, which include the consumption of less animal fat, are characterized by lower calorie consumption. In animal models, a restricted-calorie diet did not exert a significant effect on body weight, although it did reduce hyperglycemia and ventricular hypertrophy, and controlled myofiber growth following volume and pressure overload [49]. These molecular alterations observed with a calorie-restricted diet are related to lower NP levels, as pressure and level overload seem to be better handled. In a recent multicenter study including 120 malnourished patients with heart failure, a diet optimization intervention with a Mediterranean pattern was related to lower rates of all-cause mortality and resubmission for heart failure, an improvement in left ventricular ejection fraction, and decrease in NT-proBNP levels [50] compared to the control group. The additional effect of a rehabilitation program was also mentioned. The anti-inflammatory role of the Mediterranean diet has been revealed in previous population studies [51], illustrating the important role that this type of diet has on the course of HFpEF where inflammation predominates [52]. In advanced HF stages, with predominant right ventricular failure, sarcopenia and cachexia show both causality and prognostic effect on the clinical course of these patients. Nutritional support in these patients has shown to reduce inflammatory burden and NP levels, revealing a therapeutic option. This was presented in a recent study in which the NP levels were not associated with NT-proBNP, left ventricular ejection fraction, or circulating levels of cytokines [53], probably reflecting the fact that diet intervention plays a role in several pathophysiological pathways. 

The long-term consumption of n-3 fatty acids also seems to prevent age-related diastolic dysfunction; thus, is has been related to lower levels of NPs [54]. The supportive pathophysiological mechanisms involve the upregulation of the mitochondrial tricarboxylic acid enzyme Idh2 and the antioxidant enzymes SOD1 and Gpx1. N-fatty acid consumption has also been associated with reduced inflammation and extracellular matrix remodeling, with a significant downregulation of the fibrosis biomarkers MMP-2 and TGF-β in both cardiac and vascular tissues obtained from aged mice [54]. Thus, the enrichment of hypocaloric diets for cachectic heart failure patients with omega-3 fatty acid has also shown beneficial effects [50]. According to the European Society of Cardiology, the American College of Cardiology, and the American Heart Association, n-3 fatty acid consumption is recommended with an evidence level of B to reduce the risk of cardiovascular hospitalization and death [55].

Additionally, even the time of evening meal consumption seems to have impact on cardiorespiratory capacity, as later nutrient intake may help prevent the fasting-related stress associated with cardiac metabolic disturbances present in HFpEF [56].

In daily clinical practice, the identification of HFpEF is not always a straightforward process. Most of the time, patients with HF symptoms and preserved left ventricular ejection fraction are clustered into phenogroups based on their comorbidities and symptoms. In the field of research, to identify treatment modalities, many clinical models have been used to mimic HFpEF, like mouse and rat models for defining aging; dog models for arterial hypertension and renal dysfunction; and cat and pig models for cardiometabolic syndrome. Unfortunately, even these models have been unable to illustrate the reproduction of symptoms beyond morphological alteration and sometimes functional aspects of HFpEF. In these animal models, diet interventions have shown effects in some studies, although NPs have been investigated in almost half of the models [57]. Detailed differences in HFpEF pathophysiology remain unknown, and further animal studies could be more representative in terms of sex and biochemical markers to illustrate possible differences in not only the pathophysiology, but also the therapeutic role of dietary and pharmacological interventions. 

Even in a sub-analysis of IPRESERVE study (Irbesartan in Heart Failure with Preserved Ejection Fraction); although patients with a low NTproBNP had less atrial fibrillation, myocardial infarction, diabetes, chronic obstructive pulmonary disease, anemia and better renal function, their health status was similarly impaired with patients with higher NT-proBNP levels [58]. Another analysis of RELAX study revealed that higher NT-proBNP/cGMP ratio was associated with greater left ventricular mass and troponin levels, higher prevalence of atrial fibrillation, lower estimated glomerular filtration rate and peak oxygen consumption, but even this adverse cardiometabolic profile was not improved by the administration of selective phosphodiesterase-5 inhibition [59]. Those findings illustrate the complex pathophysiological procedures involved in HFpEF, while emphasizing the need for early initiation and up-titration of medications irrespectively of NTproBNP levels. 

## 9. Conclusions

Developing a standardized strategy to screen and intervene in patients at risk of HF can be difficult because of the different definitions of HF risk, the heterogeneity of prevalence in different populations, the variable duration until clinical HF or LVD develops, and the variable interventions for risk factor modification or treatment. It has become more obvious that the diagnosis of HF cannot be based on clinical signs and symptoms alone, and that markers reflecting hemodynamic conditions such as elevated NP levels can determine an HF diagnosis. Special consideration should be taken concerning HF mildly-reduced EF and HFpEF, where the diagnostic role of NPs is more limited compared to HFrEF. The modifiable role of the diet can also be further investigated in patients at various stages of HFpEF.

This scientific evidence supports the role of a healthy dietary pattern on the subclinical occurrence of heart disease as it is favorably associated with ventricular function, conferring a lower risk of heart failure. Additionally, in patients with HFpEF, the type of diet consumed may have modifying effects on plasma levels of NPs, irrespective of pharmacological treatment.

## Figures and Tables

**Table 1:** Causes of NT-proBNP elevation and echocardiographic correlations.

**Cardiac** – Heart failure, acute and chronic – Acute coronary syndromes – Atrial fibrillation – Valvular heart disease – Cardiomyopathies – Myocarditis – Cardioversion – Left ventricular hypertrophy	Echocardiographic correlations – Left atrial enlargement – Increased ratio E/e’ – Left ventricular longitudinal and circumferential strain
**Noncardiac** – Age – Renal impairment – Pulmonary embolism – Pneumonia (severe) – Obstructive sleep apnea – Critical illness – Bacterial sepsis – Severe burns – Cancer chemotherapy – Toxic and metabolic insults – Excess sodium intake	Pathophysiologic etiology for NT-proBNP elevation – Increased wall tension – Elevated filling pressures – Cardiomyocyte stretch
